# Investigating the Efficacy of Various Photosensitizers and Irradiation Strategies in Antimicrobial Photodynamic Inactivation on Different Types of Microbes

**DOI:** 10.3390/ijms27104550

**Published:** 2026-05-19

**Authors:** Lucie Válková, Markéta Kolaříková, Robert Bajgar, Renata Večeřová, Kateřina Bartoň Tománková, Hanna Dilenko, Kateřina Langová, Milan Kolář, Hana Kolářová

**Affiliations:** 1 Department of Medical Biophysics, Faculty of Medicine and Dentistry, Palacky University in Olomouc, Hněvotínská 976/3, 779 00 Olomouc, Czech Republic; 2 Department of Microbiology, Faculty of Medicine and Dentistry, Palacky University in Olomouc, Hněvotínská 976/3, 779 00 Olomouc, Czech Republic

**Keywords:** antimicrobial photodynamic therapy in vitro, antimicrobial photodynamic inactivation, photosensitizers, irradiation protocol, phototoxicity, antibacterial therapy

## Abstract

Antimicrobial photodynamic therapy is a method that utilizes photodynamic inactivation of microorganisms exposed to a photosensitizer irradiated by a specific wavelength, followed by the formation of reactive oxygen species and subsequent oxidative stress. In contrast to antibiotics, which are generally efficient against specific microorganisms, photodynamic inactivation exhibits efficacy against a wide range of bacteria, representing a promising and non-invasive alternative to treating infections caused by pathogens of different origins. This study compares the antibacterial efficacy of five different photosensitizers, including TMPyP, Protoporphyrin IX, PdTPPS_4_, Methylene Blue, and ZnPCS_2_, against eight representatives of various pathogens, including Gram-negative bacteria *Escherichia coli*, *Pseudomonas aeruginosa*, Gram-positive bacteria *Staphylococcus aureus*, *Staphylococcus epidermidis*, *Enterococcus faecalis*, *Enterococcus faecium*, *MRSA* and *Bacillus subtilis*. An optimal irradiation protocol was developed based on growth curve measurements involving double irradiation. To induce the photodynamic effect, we utilized LED emitters with wavelengths of 414 nm and 660 nm, chosen to align with the photophysical properties of the photosensitizers. Additionally, the research included assessments of the radiation’s phototoxicity and the photosensitizers’ dark toxicity against specific microorganisms. The optical properties of the photosensitizers were analyzed using absorption spectrophotometry. The effectiveness of photodynamic inactivation was assessed by determining the minimum inhibitory and bactericidal concentrations. This study aimed to identify the most suitable photosensitizer for clinical application, considering the toxicity of the photosensitizer, the radiant exposure, and its efficacy in photodynamic inactivation.

## 1. Introduction

Widespread misuse of conventional antibiotics has driven the emergence of resistant bacterial strains, posing a serious global threat and prompting intense research efforts to find effective solutions [1,2]. This issue has prompted the development of new therapeutic methods to combat multi-resistant bacterial strains [3]. There are generally two main strategies for addressing the antibacterial resistance crisis. The first focuses on developing compounds that can restore the effectiveness of existing antibiotics through synergistic interactions [4]. The second strategy seeks to identify novel antibacterial agents with high therapeutic efficacy and minimal side effects [5]. Within this framework, photosensitizing compounds have emerged as promising candidates for use in antibacterial photodynamic therapy (aPDT), a technique showing considerable potential in combating bacterial infections [6].

When a photosensitizer is exposed to light of an appropriate wavelength, it absorbs the energy and transitions to an excited singlet state, followed by intersystem crossing to a lower-energy, longer-lived triplet state. From this state, reactive oxygen species (ROS) are generated through photochemical reactions, leading to oxidative stress and microbial cell death [7,8]. ROS can be produced through two main pathways: in the Type I mechanism, the photosensitizer interacts with organic substrates to form free radicals or radical ions via electron transfer, while in the Type II mechanism, it transfers energy to molecular oxygen, producing singlet oxygen. In both cases, these reactive species damage bacterial membranes and proteins, causing leakage and cell death [9,10]. Although the effectiveness of photosensitizers is closely related to the quantum yield of ROS generation, in this study, we evaluated their efficacy primarily in terms of antibacterial activity, determined using the microdilution method according to EUCAST guidelines. The ROS-generating properties of the selected photosensitizers have been well established in the literature; therefore, the focus of this work was on their overall biological effect under standardized experimental conditions [11,12,13].

Antibacterial photodynamic therapy (aPDT) is a rapidly evolving research area that utilizes a wide range of chemically diverse photosensitizers. To date, various classes of compounds have been investigated for this purpose, including xanthene and phenothiazine dyes, porphyrin derivatives, phthalocyanines, psoralens, perylenequinonoids, and other macrocyclic molecules [9]. In the present study, the selection of photosensitizers was guided by their relevance for potential clinical application in antimicrobial photodynamic therapy. We intentionally included representatives from several major classes of photosensitizing compounds—porphyrins (TMPyP, Protoporphyrin IX, PdTPPS_4_), a phenothiazine dye (methylene blue), and a phthalocyanine (ZnPCS_2_)—to enable a systematic comparison of their photophysical properties and antimicrobial efficacy. This comparative approach was designed to identify the most suitable candidate for subsequent translational and clinical research. The main advantage of photodynamic therapy over conventional antibiotic treatments lies in its broad-spectrum activity. Photosensitizers can effectively inactivate a wide variety of microorganisms, including bacteria, protozoa, and fungi [14].

Unlike traditional antibiotics, aPDT acts independently of bacterial resistance mechanisms, offering a multi-target mode of action capable of disrupting vital cellular components and eliminating virulence factors such as enzymes and proteins. This versatility makes aPDT an effective strategy against multidrug-resistant bacteria and pathogenic biofilms. Moreover, by optimizing irradiation parameters, it is possible to maximize antimicrobial efficacy while minimizing damage to surrounding healthy tissues [15,16,17,18]. The effectiveness of aPDT varies based on the sensitivity of different bacterial species, which is influenced mainly by the structure of their cell membranes [19]. The differing structural complexity of Gram-positive and Gram-negative cell walls fundamentally influences photosensitizer uptake, with the more permeable Gram-positive barrier enabling easier PS diffusion, while the outer membrane of Gram-negative bacteria restricts access and reduces photodynamic efficacy [20,21].

This study aims to evaluate the effectiveness of various photosensitizers in antibacterial photodynamic inactivation (aPDI), specifically their ability to combat a range of pathogenic microorganisms. The focus is on assessing the antimicrobial photodynamic efficacy of different photosensitizers, including porphyrins TMPyP, Protoporphyrin IX (PP IX), PdTPPS_4_, phenothiazine dye methylene blue (MB), and zinc phthalocyanine (ZnPCS_2_), against a broad spectrum of bacteria such as *Escherichia coli*, *Pseudomonas aeruginosa*, *Staphylococcus aureus*, *Staphylococcus epidermidis*, *Enterococcus faecalis*, *Enterococcus faecium*, *MRSA* and *Bacillus subtilis*. By systematically evaluating the effectiveness of these photosensitizers, this research aims to identify the optimal conditions and parameters for successful aPDI applications. The findings are expected to contribute to the development of more effective and tailored aPDT protocols, potentially improving clinical treatment options for infections, especially those caused by multidrug-resistant pathogens.

## 2. Results

### 2.1. Irradiation Strategy

**Figure 1:**
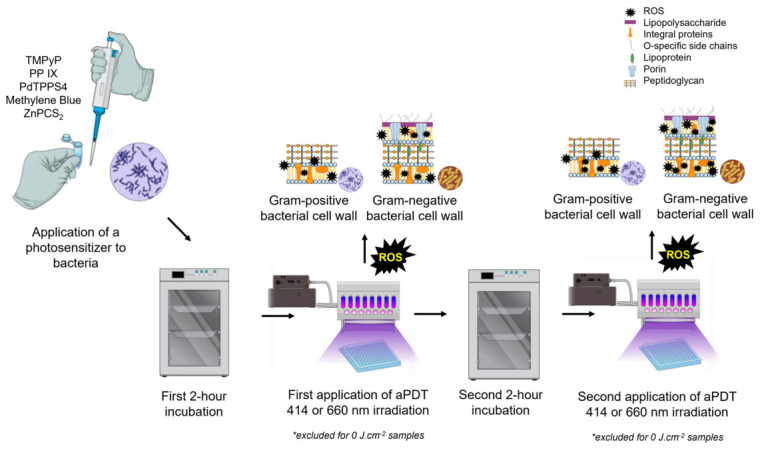
Experimental setup of antibacterial photodynamic inactivation with double irradiation step.

Two diode emitters with wavelengths of 414 nm and 660 nm, which correspond to the absorption bands of the photosensitizers, were used as light sources to induce the photodynamic effect. Various radiant exposures were established for different bacterial species and photosensitizers. A double irradiation step was implemented to enhance bacterial kill rates, minimize the risk of resistance, and improve treatment efficacy (Figure 1).

**Figure 2:**
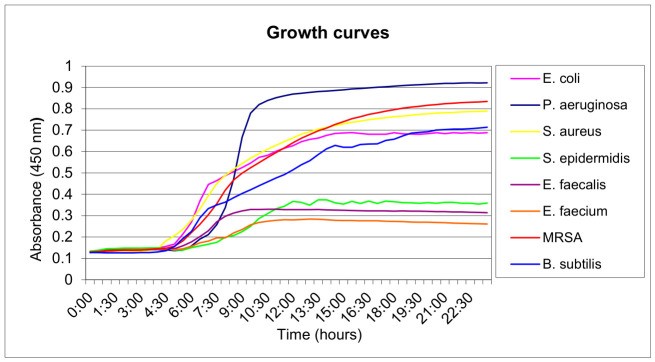
Growth curves of evaluated bacteria at 37 °C after inoculation of bacteria into a 96-well plate.

Growth curve measurements (Figure 2) showed that bacteria entered the exponential phase 4–6 h after incubation at 37 °C. Consequently, the first irradiation was conducted 2 h after incubation, before the onset of exponential growth. To increase photodynamic efficacy, a second radiant exposure was applied 2 h after the first, at the end of the lag phase.

### 2.2. Phototoxicity

The impact of light sources at 414 nm and 660 nm on bacteria can differ significantly based on the type of bacteria. This variation is primarily due to differences in their light absorption properties and the mechanisms of photodynamic action. Table 1 summarizes the maximum radiant exposures achievable with emitters at 414 and 660 nm. The initial radiant exposure of 2 × 75 J·cm^−2^ was gradually reduced to identify the highest radiation level that did not inhibit bacterial growth.

For a wavelength of 414 nm, the highest usable non-toxic radiant exposure for Gram-positive Staphylococci (including *S. aureus*, *S. epidermidis*, and *MRSA*) was found to be 2 × 10 J·cm^−2^. For Enterococci (*E. faecalis* and *E. faecium*), a radiant exposure of 2 × 20 J·cm^−2^ was suitable. A 2 × 20 J·cm^−2^ radiant exposure was non-toxic for the Gram-negative bacterium *P. aeruginosa*. In contrast, higher radiant exposures of 2 × 50 J·cm^−2^ were tolerated by another Gram-negative bacterium, *E. coli*.

For the 660 nm emitter, the toxic effects of radiation were observed only in certain bacterial strains. The maximum radiant exposure of 2 × 75 J·cm^−2^ was non-toxic to Gram-negative strains, including *P. aeruginosa* and *E. coli*, as well as to *B. subtilis*. In contrast, for Gram-positive bacteria, including *S. aureus*, *S. epidermidis*, and *MRSA*, the highest non-toxic radiant exposure was 2 × 30 J·cm^−2^. This radiant exposure was also safe for the strain *E. faecium*. However, for *E. faecalis*, a higher radiant exposure of 2 × 50 J·cm^−2^ could be applied without toxicity.

### 2.3. TMPyP

**Figure 3:**
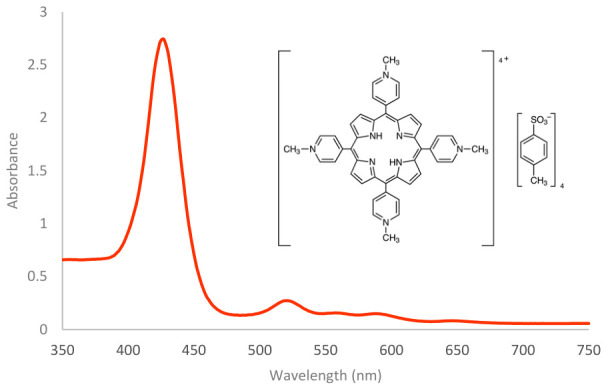
Absorption spectrum of a 50 μM TMPyP and its molecular structure.

TMPyP, or α,β,γ,δ-Tetrakis(1-methylpyridinium-4-yl)porphyrin p-Toluenesulfonate, is a cationic porphyrin and a type of tetrapyrollic organic pigment. This heterocyclic compound consists of four pyrrole rings linked by methine bridges. TMPyP is highly soluble and stable in aqueous solvents. In these solvents, it exhibits several absorption peaks, with maximum absorption at 424 nm (Figure 3). At longer wavelengths, it exhibits four weaker Q bands corresponding to minor peaks in the green and red regions (specifically at 518, 554, 585, and 630 nm) [22].

Dark toxicity was observed with TMPyP at a MIC of 50 μM for *S. aureus* in a series of tests using a two-fold dilution system. For *S. epidermidis* and *B. subtilis*, the MIC was found to be 100 μM. In contrast, no MIC was determined for the Gram-negative strains *E. coli* and *P. aeruginosa* and the Gram-positive enterococci *E. faecalis* and *E. faecium*, indicating that their MIC is higher than 200 μM. The MIC for the *MRSA* strain was 200 μM. Notably, the MIC values for dark toxicity of TMPyP were identical to the MBC values. These results suggest that at higher concentrations, TMPyP exhibits dark toxicity towards staphylococci (*S. aureus*, *S. epidermidis*, *MRSA*) and *B. subtilis*.

TMPyP demonstrated effectiveness against all nine evaluated microorganisms following irradiation, with MIC values determined for each. The highest MIC value, indicating the lowest efficacy, was observed in Gram-negative *E. coli*, where the MIC and MBC were determined at 200 μM. For *P. aeruginosa*, the MIC was 50 μM, while the MBC remained at 200 μM. Among Gram-positive bacteria, MIC/MBC values varied widely, even within the same genus. Interestingly, for the *S. aureus* strain, which exhibited higher dark toxicity and was anticipated to be more sensitive to PDT, the MIC and MBC values after irradiation were 25 μM. The MIC was 6.25 μM for the MRSA strain, and the MBC was 12.5 μM. The most significant effects were observed with *S. epidermidis*, where the MIC and MBC values were lower than 0.20 μM within the studied concentration range. In contrast, different results were noted for enterococci: for *E. faecalis*, the MIC of TMPyP was 200 μM, with an MBC greater than 200 μM. For *E. faecium*, the MIC was 12.5 μM, while the MBC was 200 μM. TMPyP also showed high efficacy against *B. subtilis*, where the MIC and MBC were observed at 0.39 μM. A summary of the results and radiant exposures for all strains can be found in Table 2. 

### 2.4. Protoporphyrin IX

**Figure 4:**
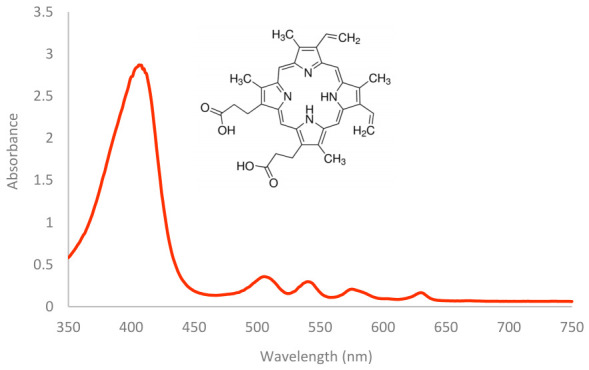
Absorption spectrum of a 50 μM PP IX and its molecular structure.

Protoporphyrin IX (3,7,12,17-Tetramethyl-8,13-divinyl-2,18-porphinedipropionic acid) is a heterocyclic organic compound comprising four pyrrole rings. As a hydrophobic substance, it is insoluble in water [23]. Additionally, PP IX is found in all human cells and is an intermediate in heme synthesis [24]. The absorption maximum of PP IX in DMSO is observed at 409 nm, corresponding to the Soret band. Additionally, four other absorption bands (Q bands) are present in the visible spectrum at 510, 544, 578, and 635 nm (see Figure 4). In aqueous media, PP IX partially aggregates, causing a red shift and reduced absorption [25].

No dark toxicity of PP IX was found in any of the studied strains, even at concentrations exceeding the highest level assessed, which was 200 µM. The results indicate that PP IX was ineffective against the Gram-negative bacteria *E. coli* and *P. aeruginosa*, which had MICs greater than 200 µM. In contrast, significant effects were observed after irradiation in all seven studied strains of Gram-positive bacteria.

Among the staphylococci, PP IX was most effective against *S. epidermidis*, with the MIC and MBC recorded at 0.20 µM. A similar result was found for *S. aureus*, which also had identical MIC and MBC values of 0.78 µM. For the MRSA strain, the MIC and MBC values were 12.5 µM.

In terms of enterococci, *E. faecalis* showed higher efficacy with an MIC of 0.39 µM and an MBC of 3.13 µM, compared to *E. faecium*, which had both MIC and MBC values of 3.13 µM—this was also the case for *B. subtilis*. A summary of the results and radiant exposures can be found in Table 3.

### 2.5. PdTPPS_4_

**Figure 5:**
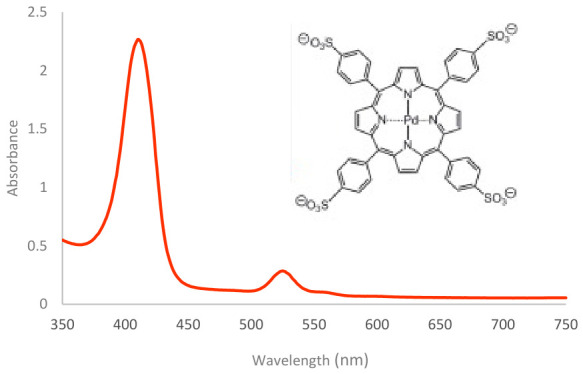
Absorption spectrum of a 50 μM PdTPPS_4_ and its molecular structure.

PdTPPS_4_ (5,10,15,20-tetrakis (4-sulfonatophenyl) porphyrin-Pd(II)) is a water-soluble palladium metalloporphyrin with a conjugated tetrapyrrole macrocycle. The central metal ion significantly affects the photophysical properties of metalloporphyrins [26]. Its sulfonate groups ensure the high aqueous solubility required for biological applications [27]. The absorption spectrum of PdTPPS_4_ features a Soret band around 410 nm and a second absorption peak at 530 nm, corresponding to the Q band transition, similar to that observed in PP IX (Figure 5).

The highest level of dark toxicity was observed with PdTPPS_4_ in *S. epidermidis*, where the MIC was 50 μM, and the MBC was 200 μM. In the case of *S. aureus*, both the MIC and MBC were determined at 200 μM. *E. faecium* also showed MIC of 200 μM, but this concentration was not bactericidal, as the MBC was greater than 200 μM. For all other strains, the MIC values were not found within the range of evaluated concentrations and are likely higher than 200 μM.

The study found that PdTPPS_4_ was ineffective against *E. coli* and *B. subtilis* after PDT, with the MIC and MBC exceeding 200 μM. For *P. aeruginosa*, the MIC was observed at 50 μM, but no MBC was established (greater than 200 μM). In contrast, all studied Gram-positive bacteria, except for *B. subtilis*, showed measurable MIC values. *S. epidermidis* exhibited the best results among staphylococci, with MIC and MBC at or below the lowest assessed concentration of 0.20 μM. For *S. aureus* and *MRSA*, the MIC was 12.5 μM; this concentration was bactericidal for *S. aureus*, while the MBC for *MRSA* could not be determined (greater than 200 μM). The enterococci *E. faecalis* and *E. faecium* had a MIC of 25 μM. Notably, for *E. faecium*, the MIC was equal to the MBC, while for *E. faecalis*, the MBC could not be determined (greater than 200 μM). The results and radiant exposures are summarized in Table 4.

### 2.6. Methylene Blue

**Figure 6:**
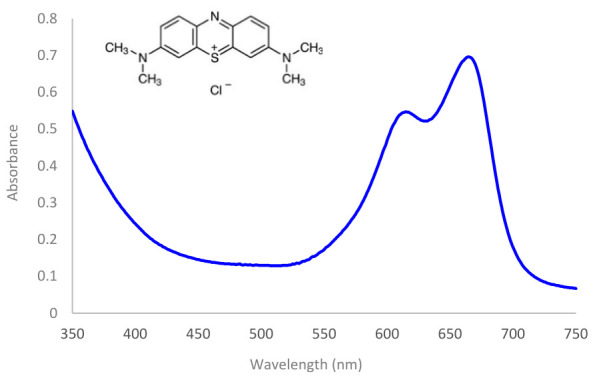
Absorption spectrum of a 50 μM Methylene blue and its molecular structure.

MB, a member of the phenothiazinium class of compounds, is a widely used heterocyclic cationic dye. As a first-generation photosensitizer, it binds efficiently to both Gram-positive and Gram-negative bacteria and is widely used in aPDT [28]. At room temperature, MB appears as a solid, odorless, dark green powder. In aqueous solution, MB forms stable, highly soluble monomeric and dimeric species. It is highly soluble in water, allowing for the formation of stable solutions at room temperature. Additionally, MB is soluble in various organic solvents, including methanol, 2-propanol, ethanol, acetone, and ethyl acetate. MB exhibits a strong absorption band in the 550 to 700 nm range, with a peak absorption maximum of 664 nm associated with the MB monomer. There is also a shoulder peak around 612 nm attributed to the MB dimer (Figure 6). The absorption spectrum of MB varies with concentration due to dimerization; this process is influenced by ionic strength and can be affected by the presence of charged interfaces [29].

The dark toxicity of MB was not observed in the Gram-negative bacteria *E. coli* and *P. aeruginosa*, as they both had MIC and MBC values greater than 200 µM. However, MIC values were determined for all other studied pathogens. For staphylococci, the MIC was 100 µM for both *S. aureus* and *MRSA*, but this concentration was not bactericidal, with MBC values exceeding 200 µM. In contrast, for *S. epidermidis*, the MIC was 12.5 µM, and the MBC was 50 µM. In the case of enterococci, *E. faecalis* and *E. faecium* had an MIC of 200 µM. For *E. faecalis*, this concentration was bactericidal (MBC = 200 µM), while for *E. faecium*, it was not (MBC > 200 µM). For *B. subtilis*, the MIC was observed at 50 µM, with an MBC of 100 µM.

Despite its higher dark toxicity, MB produced excellent results after PDT in all studied strains. This included Gram-positive *E. coli* and *P. aeruginosa*, which showed no effects from porphyrins (TMPyP, PP IX, and PdTPPS_4_) within the evaluated concentration range after PDT.

The MIC and MBC values were assessed for various microorganisms. For *E. coli*, the concentration was set at 6.25 μM. For *P. aeruginosa*, the MIC was found to be 1.56 μM, with an MBC of 3.13 μM. In the case of *S. aureus*, both the MIC and MBC were measured at 0.20 μM, while for *S. epidermidis* and *MRSA*, these values were 0.39 μM. In the case of enterococci, *E. faecalis* had MIC and MBC values of 6.25 μM, whereas *E. faecium* showed an MIC of 12.5 μM and an MBC of 25 μM. The bacterium *B. subtilis* exhibited both MIC and MBC values of 3.13 μM. MB demonstrated high efficacy against all assessed microorganisms, showing activity at very low concentrations, even against generally more resistant Gram-negative strains. The concentrations that inhibited growth were also bactericidal, except for *E. faecium* and *P. aeruginosa*, where the MBC was one dilution higher than the MIC. Results and radiant exposures for all strains can be found in Table 5.

### 2.7. Disulphonated Zinc Phthalocyanine

**Figure 7:**
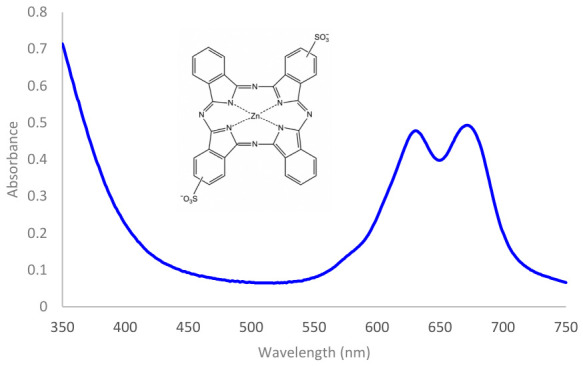
Absorption spectrum of a 50 μM ZnPCS_2_ and its molecular structure.

Disulphonated zinc phthalocyanine (ZnPCS_2_), synthesized by Jiri Mosinger, exhibits absorption in the range of 550 to 730 nm [30]. It has two prominent absorption peaks, known as Q bands at 630 nm and 670 nm (Figure 7). The two sulfone groups markedly improve the aqueous solubility of zinc phthalocyanine.

In the assessed concentration range, the dark toxicity of ZnPCS_2_ was observed only in *S. epidermidis* (minimum inhibitory concentration, MIC = 50 μM) and *MRSA* (MIC = 100 μM). However, the inhibitory concentration in both strains did not exhibit bactericidal activity, as the minimum bactericidal concentration (MBC) was greater than 200 μM. Following photodynamic inactivation, ZnPCS_2_ demonstrated positive efficacy against all studied microbial strains. For Gram-negative *E. coli*, both the MIC and MBC reached 100 μM. In contrast, *P. aeruginosa* showed a MIC of 3.13 μM and an MBC of 6.25 μM. Similar effects of ZnPCS_2_ were observed across all three staphylococcal strains. For *S. aureus*, both the MIC and MBC were observed at 0.78 μM. In the cases of *S. epidermidis* and *MRSA*, the MIC was 0.39 μM, and the MBC was 0.78 μM for both strains. The efficacy of ZnPCS_2_ was also notable in Enterococcus species, with MIC and MBC values of 0.78 μM for *E. faecalis* and 1.56 μM for *E. faecium*. Additionally, *B. subtilis* exhibited high efficacy with MIC and MBC values at 0.78 μM. After PDT, ZnPCS_2_ was bactericidal at low concentrations against all microorganisms except *E. coli*. Results are summarized in Table 6.

## 3. Discussion

### 3.1. Repeated Irradiation Step

To enhance the effects of PDT, double irradiation was applied to microbial strains using patented LED emitters that provide uniform irradiation. Repeated irradiation enhances the cumulative production of ROS and improves the overall effectiveness of bacterial inactivation. Initial irradiation causes photosensitizer activation, producing ROS and subsequent oxidative stress, compromising the integrity of bacterial cell membranes and other cellular structures. Subsequent irradiation further activates the photosensitizer, producing additional ROS, cumulating, and enhancing overall oxidative stress. Each subsequent irradiation can penetrate deeper and cause more extensive damage to bacterial cells, especially those that survived the initial exposure [7,31]. The positive and enhancing effects of repeated irradiation have also been previously studied in anticancer therapies. For instance, repeated irradiation with a prefixed time interval in one therapy session induced necrosis to a depth 3 times greater than PDT alone [32]. De Vijlder et al. demonstrated a significant increase in the complete response rate of superficial basal cell carcinoma, following ALA-PDT using an illumination scheme in which two light fractions of 20 and 80 J/cm^2^ were delivered 4 and 6 h after the application of ALA, compared with traditional illumination [33].

### 3.2. Phototoxicity

Our study experimentally determined the maximum radiant exposure for pathogens, such as bacteria and bacilli, that do not adversely affect bacterial growth. These values varied based on the specific bacterial strain and the wavelength of the radiation source. Identifying the maximum radiant exposure without a photosensitizer makes it possible to reduce the concentration of the photosensitizer used, thereby minimizing potential side effects in in vivo applications. As the wavelength of radiation increases, the required radiant exposure also increases. The results of this study indicate that blue light alone can have phototoxic effects when used at higher radiant exposures. Specifically, blue light at a wavelength of 414 nm can be absorbed by natural compounds called porphyrins and flavins found in bacterial cells. This absorption leads to the production of ROS, which can damage and kill the cells.

#### 3.2.1. Blue Light

Blue light at 414 nm is particularly effective against Gram-positive bacteria, such as *S. aureus* and *S. pneumoniae*. These bacteria have higher levels of endogenous porphyrins, allowing them to absorb blue light efficiently and generate ROS, resulting in effective bacterial inactivation [34,35]. Blue light can also enhance the antibacterial photodynamic therapy on biofilm-forming *S. aureus*. Combining blue light (405 nm) and aPDT (810 nm combined with a photosensitizer ICG) significantly improved the bactericidal effect. However, in this study, the role of repeated exposure to blue light was also investigated, proving that biofilm showed adaptation when repeated single-wavelength antibacterial light treatment was applied after 24 h. This statement further supports the idea that repeated exposure to light may enhance antimicrobial efficacy, suggesting that repeated irradiation can strengthen the overall photodynamic therapy (PDT) effect. The response to the repeated adverse environmental stimuli seemed to develop in the early stage, within the first few repeated exposures [36]. In our study, the second irradiation was performed 2 h after the first one, shortening the time bacteria could develop potential adaptive mechanisms. In contrast, Gram-negative bacteria like *E. coli* and *P. aeruginosa* are generally more resistant to blue light due to their outer membrane barrier. However, higher radiant exposures of blue light or combining it with other treatments can still significantly reduce these bacteria [34,35]. For example, a recent study on the inactivation of *E. coli* using different light sources revealed that the blue LED at 418 nm was more effective in bacterial inactivation than the blue laser diode at 405 nm. Similarly, with its broad emission band, the red LED at 630 nm proved more efficient in inactivating bacteria than the red laser diode at 635 nm [37].

#### 3.2.2. Red Light

Red light is typically more effective for photodynamic therapy than blue light because it penetrates the skin more deeply and has no known toxicity [38]. Additionally, red light is frequently used in regenerative medicine to support healing wounds and skin conditions by stimulating biological processes, promoting healing and regeneration [39,40]. In the last 50 years, red light therapies have been extensively investigated and successfully applied in the treatment of various conditions such as wound healing, skin diseases, traumatic brain injuries, Alzheimer’s and Parkinson’s disease, depression, and inflammatory diseases [41,42]. On the other hand, increased exposure to red light can raise temperatures, potentially leading to heat shock stress in bacteria. This stress may result in inhibition that is unrelated to light’s effects.

### 3.3. Dark Toxicity of the Photosensitizers

Determining the dark toxicity of photosensitizers is essential to ensure their safety and effectiveness in photodynamic therapy (PDT) and antimicrobial treatments, as it represents their cytotoxic effects without light activation due to chemical interactions or residual ROS generation. It provides insight into potential off-target effects and helps guide appropriate dosing and administration strategies.

#### 3.3.1. Dark Toxicity of TMPyP

The dark toxicity of TMPyP was observed in several studied strains with varying minimum inhibitory concentrations: *S. aureus* (MIC = 50 µM) showed higher sensitivity than *S. epidermidis* and *B. subtilis* (MIC = 100 µM), while *MRSA* had the highest MIC at 200 µM. However, the dark toxicity of TMPyP may be affected by residual light present during experiments. This issue could be mitigated by adding substances that quench any leftover reactive oxygen species (ROS). The toxic effect of TMPyP on different types of bacteria was described earlier, but experiments differed in their experimental setup, hampering a direct comparison of the obtained data [43]. In the case of Gram-negative strains (*E. coli*, *P. aeruginosa*) and enterococci *(E. faecalis*, *E. faecium*), no significant inhibitory effect of TMPyP was observed, with a MIC greater than 200 µM. This can be explained by Gram-negative bacteria’s more complex cell wall structure, which includes an outer layer composed of lipopolysaccharides and proteins and the peptidoglycan layer. This outer layer prevents the substance from penetrating the intracellular space of the bacteria, unlike the thicker peptidoglycan layer found in Gram-positive bacteria. For these two types of Gram-negative bacteria, no MIC was determined within the evaluated range of concentrations, indicating that dark toxicity was not demonstrated for any of the studied photosensitizers (TMPyP, PP IX, PdTPPS_4_, MB, ZnPCS_2_), all showing MIC values greater than 200 µM.

#### 3.3.2. Dark Toxicity of Methylene Blue

In the case of MB, similar to TMPyP, higher dark toxicity was observed, except for certain Gram-negative strains like *E. coli* and *P. aeruginosa*, as well as in enterococci such as *E. faecalis* and *E. faecium*, which had a MIC value of 200 μM. Among the staphylococci, besides the previously mentioned *S. epidermidis*, the MIC values for *S. aureus* and *MRSA* strains were found to be 100 μM. The dark toxicity of MB has been discussed in several studies [44,45,46]. Previous research indicates that the antibacterial action of MB is primarily attributed to its unique redox properties, which disrupt various electron transport pathways in bacteria [46]. MB is also known for significant dimerization in solution as its concentration increases. Dimers enable MB to localize in different cellular regions and utilize different mechanisms of cell destruction [45]. It was found that the antifungal effect of MB is mediated through mitochondrial dysfunction and disruption of redox and membrane homeostasis, and seems to be independent of the significant drug efflux pump transporter activity, which is commonly involved in resistance mechanisms in both bacteria and fungi [47].

#### 3.3.3. Dark Toxicity of PPIX, PdTPPS_4_ and ZnPCS_2_

In the case of PP IX, no dark toxicity or inhibitory effects were observed in any of the eight evaluated pathogens, as the MIC was greater than 200 μM for all strains. The low dark toxicity of PdTPPS_4_ was also confirmed, as no MIC value of 200 μM or less was observed for any of the assessed pathogens, except for *S. epidermidis*, which had a MIC of 50 μM. The dark toxicity of photosensitizers against Staphylococci, including *S. aureus*, *S. epidermidis*, and *MRSA*, showed considerable variability, with no clear trends emerging. However, *S. epidermidis* exhibited a higher sensitivity to all studied photosensitizers except for protoporphyrin IX (PP IX). Specifically, MIC for *S. epidermidis* was highest for MB at 12.5 μM, followed by ZnPCS_2_ and PdTPPS_4_ at 50 μM and TMPyP at 100 μM. Notably, *S. epidermidis* and *MRSA* were the only strains demonstrating dark toxicity to the ZnPCS_2_ sensitizer, with MIC values of 50 μM for *S. epidermidis* and 100 μM for *MRSA*. In contrast, ZnPCS_2_ did not exhibit any inhibitory effects on other investigated strains, including *E. coli*, *P. aeruginosa*, *S. aureus*, *E. faecalis*, *E. faecium* or *B. subtilis*.

#### 3.3.4. Dark Toxicity and Its Implications for Therapeutic Dosing

The MBC of photosensitizers against the evaluated strains was less than 200 μM in only a few instances. This was explicitly observed for TMPyP and MB. In contrast, PP IX, PdTPPS_4_, and ZnPCS_2_ showed MBC values of ≥200 μM for all eight analyzed microorganisms. Understanding dark toxicity is essential for optimizing dosages and administration methods. Photosensitizers with high dark toxicity require lower concentrations to minimize off-target effects. In contrast, those with low dark toxicity are ideal, as they cause minimal harm to healthy tissues before light activation. In summary, assessing dark toxicity ensures safe and effective photosensitizers, enabling precise therapeutic control while protecting non-target tissues.

### 3.4. Antimicrobial Photodynamic Inactivation

#### 3.4.1. Blue Light Activated Porphyrins (TMPyP, PP IX, PdTPPS_4_)

In the evaluated concentration range, all five photosensitizers showed clear inhibitory—primarily bactericidal—effects after PDT with double irradiation, with the exception of PP IX and PdTPPS_4_, which displayed limited activity against selected Gram-negative strains. Overall, porphyrins were less effective against Gram-negative bacteria, whereas TMPyP emerged as the most potent photosensitizer in this group. Its activity against *E. coli* and *P. aeruginosa* can be further enhanced by co-administration with the cationic polymer Eudragit^®^ E100, which improves membrane permeability and supports deeper photosensitizer uptake, thereby strengthening PDT efficiency [48]. Recent studies also indicate that TMPyP may undergo strain-dependent reductive transformation, behaving as a photothermal agent in hypoxic environments with facultative anaerobes, while retaining classical photodynamic activity under aerobic conditions [49].

In contrast, PdTPPS_4_ showed minimal activity toward some Gram-negative strains, although partial inhibition of *P. aeruginosa* was observed. The charge and charge distribution of porphyrins are known to correlate with their bactericidal potential, highlighting the structural determinants of PDT action. Concentrations above 200 μM were not tested due to the risk of aggregation and reduced light penetration, both of which could diminish PDT efficacy [50,51]. The reduced susceptibility of Gram-negative bacteria is consistent with their outer membrane rich in lipopolysaccharides, which are negatively charged and hydrophobic [52]. This membrane blocks penetration of neutral or anionic porphyrins, whereas cationic porphyrins (e.g., TMPyP) or those conjugated to polycationic moieties overcome this uptake barrier by binding to lipopolysaccharides [53]. Cationic porphyrins, or porphyrins conjugated to polycationic structures, overcome this barrier through electrostatic interactions. PDT outcomes can also be improved by membrane-disrupting agents (e.g., PMBN, EDTA) [53] or by optimizing photosensitizer selectivity. For PP IX, using a light source overlapping multiple absorption bands may further improve activity toward *E. coli* [37].

All three porphyrins demonstrated very strong bactericidal activity against *S. epidermidis*, with effective killing at the lowest tested concentrations, a pattern similarly observed for MB and ZnPCS_2_. Given the clinical relevance of *S. epidermidis* as a biofilm-forming pathogen and the accessibility of infected sites to light, its high sensitivity to PDT is particularly significant [54].

Against *S. aureus*, PP IX and ZnPCS_2_ showed the strongest inhibition, while MB demonstrated the highest overall potency. Differences among photosensitizers may reflect their distinct interactions with bacterial membranes and intracellular targets. Previous work by Muehler et al. demonstrated concentration-dependent accumulation of TMPyP and MB in planktonic *E. coli* and *S. aureus*, with higher TMPyP and lower MB uptake in *S. aureus*. Both photosensitizers caused membrane damage in both bacteria. MB reduced bacterial replication at lower concentrations than those causing membrane damage, indicating additional mechanisms. In contrast, TMPyP primarily targeted the cell membrane even at low concentrations [55].

For *MRSA*, all three porphyrins showed comparable activity, though MB and ZnPCS_2_ were effective at sub-micromolar concentrations. Several studies have shown that *MRSA* can be effectively inactivated by photodynamic therapy (PDT) using different photosensitizers and appropriate light wavelengths. Although *MRSA* is generally more resistant to photoinactivation than antibiotic-sensitive strains, this resistance is not related to classical antimicrobial resistance mechanisms [56,57]. The effects of various photosensitizing agents in antibacterial photodynamic therapy on the *MRSA* strain are also demonstrated in a review article by Dharmaratne et al. [58]. For example, TMPyP combined with short, intense light pulses (100 ms flashes over 10 s) produced rapid and dramatic bacterial killing, including *MRSA*, *S. aureus*, *B. atrophaeus*, and *E. coli.* The generated reactive oxygen species caused oxidative damage, achieving up to a 6 log_10_ (99.9999%) reduction in bacterial viability within just 10 s at photosensitizer concentrations of 1–100 µM and radiant exposures of 20–80 J/cm^2^ [59]. Our results demonstrate that MB and ZnPCS_2_ can inactivate *MRSA* at sub-micromolar concentrations, highlighting an even higher potency than previously reported for TMPyP and other porphyrins. This underscores the potential of these photosensitizers for rapid and efficient photodynamic inactivation, confirming and extending previous findings that *MRSA* can be effectively targeted by PDT despite its generally higher resistance compared to antibiotic-sensitive strains.

PP IX was the most effective porphyrin against enterococci, while PdTPPS_4_ and TMPyP showed lower activity, reflecting differences in porphyrin–cell wall interactions and uptake in these structurally robust Gram-positive cocci. With the exception of PdTPPS_4_, which showed limited activity against *B. subtilis*, TMPyP and PP IX exhibited high bactericidal efficacy. For PdTPPS_4_, this suggests either insufficient potency at lower concentrations or a requirement for substantially higher radiant exposures.

In general, porphyrins induce microbial death through multi-target oxidative mechanisms, including functional damage (enzyme inactivation, protein oxidation, impaired DNA synthesis), morphological alteration (disruption of membrane structures essential for replication), and cell membrane destruction, leading to loss of cellular integrity. Although DNA damage may occur, bacteria can sometimes repair it, underscoring the importance of membrane and protein damage as primary bactericidal pathways in PDT [6,10,29].

#### 3.4.2. Red-Light Activated Methylene Blue and ZnPCS_2_

MB demonstrated broad-spectrum inhibitory effects against all eight tested microorganisms, with bactericidal concentrations generally in the low micromolar range. Gram-negative bacteria such as *E. coli*, *P. aeruginosa*, and *B. subtilis* were susceptible at slightly higher concentrations, while *E. faecalis* and *E. faecium* were inhibited at lower micromolar levels. Studies have shown that fractionated light exposure and repeated photosensitizer renewal can enhance MB-mediated photodynamic inactivation (PDI), highlighting the importance of irradiation strategy and photosensitizer properties for optimal efficacy [60,61].

Staphylococci, including *S. aureus*, *S. epidermidis*, and *MRSA*, were highly sensitive to MB, with effective concentrations in the sub-micromolar range. Previous studies have explored the effects and mechanisms of MB against these microorganisms, indicating that a key mechanism involves damaging the integrity of cell walls, DNA, and membrane proteins [62,63,64,65,66]. Miyabe et al. evaluated the effect of PDT with MB on 20 Staphylococcus strains isolated from the human oral cavity, including *S. aureus*, *S. schleiferi*, *S. epidermidis*, *S. capitis*, *S. haemolyticus*, and *S. lentus*. PDT effectively reduced the number of viable cells of all clinical Staphylococcus isolates studied. However, a very high concentration of MB (3 mM) was used [67]. According to our findings and other studies, the key advantage of MB is its broad-spectrum efficacy against Gram-negative and Gram-positive bacteria. Another advantage of MB is its lower cost compared to other photosensitizers and minimal toxicity to mammalian cells [68,69].

The phthalocyanine derivative ZnPCS_2_ also showed strong photodynamic activity, particularly against Gram-negative bacteria, where it was more effective than the studied porphyrins. It was highly potent against staphylococci and enterococci, with MIC and MBC values comparable to MB, and also demonstrated activity against *B. subtilis*. This synthesized photosensitizer was previously used, for example, in anticancer in vitro PDT by Pola et al. [30] also seems to be a promising antibacterial agent.

#### 3.4.3. Challenges in Comparing Antimicrobial PDT and Our Systematic Approach

Despite extensive research on antimicrobial PDI, comparing studies is challenging due to variations in experimental conditions, such as light source, photosensitizer concentration, incubation times, and solvents [70]. High photosensitizer concentrations may lead to aggregation, reducing reactive oxygen species generation and photodynamic efficiency. Additionally, differences in solubility and cell membrane permeability can affect activity [50]. Another drawback may be that traditional photosensitizers often exhibit low bioavailability: hydrophobic variants aggregate intracellularly, causing aggregation-induced quenching. In contrast, hydrophilic ones with high water solubility struggle to cross lipophilic cell membranes [71]. Although many publications are focusing on aPDT using organic photosensitizers, it is challenging to compare their efficacy due to various testing parameters, including different light sources, concentration, incubation time, a wide range of solvents, pre-irradiation times, irradiation times, radiant exposures, etc. Our research presents an innovative approach to photodynamic inactivation using five different photosensitizers against a wide range of pathogens. Importantly, it provides a systematic evaluation of five photosensitizers under consistent experimental conditions, including standardized concentrations and light wavelengths, following EUCAST and ISO guidelines. This approach allows reliable comparison of antimicrobial efficacy and offers valuable insights into the potential of photodynamic therapy against a wide range of pathogens [72,73].

Table 7 summarizes the comparative antimicrobial efficacy of the evaluated photosensitizers based on MIC and MBC values obtained under standardized experimental conditions. A clear trend can be observed, with red-light-activated photosensitizers (methylene blue and ZnPCS_2_) demonstrating superior and more consistent broad-spectrum activity, including against Gram-negative bacteria. In contrast, porphyrin-based photosensitizers showed variable efficacy, with reduced activity primarily against Gram-negative strains, likely due to limited cellular uptake.

## 4. Materials and Methods

### 4.1. Bacterial Strains

For this study, the microorganisms used were *Escherichia coli* CCM 3954, *Pseudomonas aeruginosa* CCM 3955, *Staphylococcus aureus* CCM 4223, *Staphylococcus epidermidis* CCM 7221, *Enterococcus faecalis* CCM 4224 obtained from the Czech Collection of Microorganisms (Masaryk University, Brno), as well as Methicillin-oxacilin resistant *Staphylococcus aureus* 4591 (MRSA), Vancomycin-resistant *Enterococcus faecium* 419 (VRE) and *Bacillus subtilis* 168 obtained from the microorganism collection of the Department of Microbiology at the Faculty of Medicine of Palacký University in Olomouc. The results of antibiotic susceptibility in resistant MRSA and VRE strains are summarized in Table 8. Strains were cultured on blood agar for 24 h, Mueller–Hinton (MH) medium was utilized for experiments.

### 4.2. Photosensitizers

Commercially available photosensitizers Methylene Blue (Thermo Fisher Scientific, Waltham, MA, USA, high purity), α,β,γ,δ-Tetrakis(1-methylpyridinium-4-yl)porphyrin p-Toluenesulfonate (Tokyo Chemical Industry Co., Ltd., Tokyo, Japan, ≥98.0%), Protoporphyrin IX (Sigma-Aldrich, St. Louis, MO, USA, ≥95%) and 5,10,15,20-tetrakis (4-sulfonatophenyl) porphyrin-Pd(II) (Porphyrin Systems, Lüebeck, Germany, 98%) were used in the study, as well as synthesized photosensitizers from Jiri Mosinger disulphonated zinc phtalocyanine. Further, 10 mM stock solutions of the photosensitizers were prepared by dissolving powdered forms of photosensitizers in 1 mL of PBS (MB, TMPyP, ZnPCS_2_, PdTPPS_4_) or 1 mL of DMSO (PP IX).

### 4.3. Absorption Spectroscopy

Photosensitizers were characterized using absorption spectrometry with a Synergy HT multi-function plate reader (BioTek^®^ Instruments, Inc., Winooski, VT, USA). To prepare the samples, 50 µM solutions of the photosensitizers were made by dissolving them in phosphate-buffered saline (PBS) for MB, TMPyP, ZnPCS_2_, and PdTPPS_4_, while protoporphyrin IX (PP IX) was dissolved in dimethyl sulfoxide (DMSO). The absorption spectra of photosensitizers were measured over a wavelength range of 350 to 750 nm with a step size of 1 nm.

### 4.4. Microbial Growth Measurement (Growth Curves)

To determine the optimal irradiation protocol based on microbial growth, turbidity (absorbance) was measured over time on a plate multifunction reader to determine growth curves. Microbes cultured overnight on MH-agar were resuspended in 2 mL of saline to a McFarland optical density of 1. Bacteria were then inoculated at 5 × 10^5^ into a flat-bottom plate containing 100 μL of MH medium/well, covered with cellophane foil to eliminate evaporation and placed in the reader for 24 h at 37 °C. Absorbance (turbidity) was measured at a wavelength of 450 nm, from which growth curves were subsequently constructed as a function of time.

### 4.5. Microbial Cultivation and Dark Toxicity of the Photosensitizers

Bacterial strains *Staphylococcus aureus*, *MRSA*, *Staphylococcus epidermidis*, *Enterococcus faecalis*, *Enterococcus faecium*, *Escherichia coli*, *Pseudomonas aeruginosa* and *Bacillus subtilis* were incubated on blood agar for 24 h. Subsequently, a bacterial suspension was prepared in saline, where the turbidity or concentration of bacteria was determined using a densitometer to an optical density of McFarland 1, corresponding to 5 × 10^8^ CFU/mL. Stock solutions of photosensitizers prepared in PBS (in DMSO for PP IX) were diluted in microtiter plates in MH-culture medium using a two-fold dilution system in a concentration series (200; 100; 50; 25; 12.5; 6.25; 3.13; 1.56; 0.78; 0.39; 0.20 μM). The plates were then inoculated with microbial suspensions to a final bacterial concentration of 5 × 10^5^ CFU/mL per well. Appropriate control wells were included in each microdilution assay in accordance with EUCAST recommendations. The growth (positive) control consisted of bacterial suspension in Mueller–Hinton medium without photosensitizer, which confirmed the viability of the tested strains and suitability of the growth conditions, as evidenced by consistent bacterial growth in all experiments. A sterility (negative) control consisting of sterile medium without bacterial inoculum was included to verify the absence of contamination. The plates thus prepared were placed in a thermostat for two hours, and then were ready for further testing.

### 4.6. Photodynamic Inactivation and Phototoxicity

After preparation and 2 h incubation in a thermostat, bacteria exposed to various photosensitizers were irradiated with specific radiant exposures concerning the bacterial strain by a light emitting diode (LED) source emitting light of 414 ± 20 nm (made of 525 LED elements ATI-2835SUV02-C1, Asiatech Incorporation Limited, China) or 660 ± 20 nm (made of 980 LED elements KP-1608SRC-PRV, Kingbright, China) with a wavelength corresponding to the absorption maximum of the photosensitizer used. To induce a photodynamic effect, TMPyP, PPIX, and PdTPPS_4_ were irradiated with an emitter with a wavelength of 414 nm at a current of 2 A for irradiation of photosensitizers MB and ZnPCS_2_; an emitter with a wavelength of 660 nm at a current of 3 A was used. For the 660 nm wavelength, an exposure time of 77 s was required to achieve a radiant dose of 1 J/cm^2^, whereas for the blue 414 nm light source, an exposure time of 13 s was required to achieve the same radiant dose of 1 J/cm^2^. The radiant exposures used varied for specific bacteria, depending on the phototoxicity of the radiation, and are summarized in Table 1 and discussed in the results section.

### 4.7. Determination of MIC and MBC

Antibacterial activity was assessed by determining minimum inhibitory concentration (MIC) and minimum bactericidal concentration (MBC) using a standard microdilution method according to EUCAST recommendations (The European Committee on Antimicrobial Susceptibility Testing—EUCAST). The MIC value was determined to evaluate the effectiveness of photodynamic inactivation and the dark toxicity of the photosensitizer, and was recorded 24 h after the bacteria were first exposed to the photosensitizers. The MIC represents the lowest concentration of photosensitizer that visibly inhibits bacterial growth. To assess the MBC of the photosensitizers, the contents of wells with visibly inhibited growth were inoculated onto blood agar and incubated at 35 °C for 24 h. The MBC was defined as the lowest concentration of photosensitizer, resulting in no visible growth of microbial colonies.

## 5. Conclusions

Our findings highlight key methodological and photophysical parameters that can be leveraged to optimize aPDT and tailor its application to specific pathogens. By demonstrating that repeated irradiation substantially amplifies oxidative stress and bacterial inactivation, this study offers a clear pathway toward developing more robust and clinically relevant photodynamic strategies. These results lay important groundwork for improving the management of infections—particularly those caused by multidrug-resistant microorganisms—and reinforce the growing potential of aPDT as a complementary or alternative approach to conventional antibiotic therapy.

## Figures and Tables

**Table 1:** Used radiant exposures of emitters with wavelengths of 414 nm and 660 nm for individual bacteria.

	414 nm	660 nm
*Escherichia coli*	2 × 50 J·cm^−2^	2 × 75 J·cm^−2^
*Pseudomonas aeruginosa*	2 × 20 J·cm^−2^	2 × 75 J·cm^−2^
*Staphylococcus aureus*	2 × 10 J·cm^−2^	2 × 30 J·cm^−2^
*Staphylococcus epidermidis*	2 × 10 J·cm^−2^	2 × 30 J·cm^−2^
*Enterococcus faecalis*	2 × 20 J·cm^−2^	2 × 50 J·cm^−2^
*Enterococcus faecium*	2 × 20 J·cm^−2^	2 × 30 J·cm^−2^
*MRSA*	2 × 10 J·cm^−2^	2 × 30 J·cm^−2^
*Bacillus subtilis*	2 × 10 J·cm^−2^	2 × 75 J·cm^−2^

**Table 2:** MIC and MBC values of TMPyP on different pathogens without irradiation (dark toxicity) and after PDT. The color scale indicates the relative therapeutic effectiveness within the tested concentration range, with green corresponding to the highest effectiveness and red corresponding to the lowest effectiveness.

Bacteria	Dark Toxicity		After PDT		
	MIC (μM)	MBC (μM)	Irradiance (J·cm^−2^)	MIC (μM)	MBC (μM)
*Escherichia coli*	>200	>200	2 × 50	200.00	200.00
*Pseudomonas aeruginosa*	>200	>200	2 × 20	50.00	>200
*Staphylococcus aureus*	50	50	2 × 10	25.00	25.00
*Staphylococcus epidermidis*	100	100	2 × 10	<0.2	<0.2
*Enterococcus faecalis*	>200	>200	2 × 20	200.00	>200
*Enterococcus faecium*	>200	>200	2 × 20	12.5	200
*MRSA*	200	200	2 × 10	6.25	12.50
*Bacillus subtilis*	100	100	2 × 10	0.39	0.39

**Table 3:** MIC and MBC values of PP IX on different pathogens without irradiation (dark toxicity) and after PDT. The color scale indicates the relative therapeutic effectiveness within the tested concentration range, with green corresponding to the highest effectiveness and red corresponding to the lowest effectiveness.

	Dark Toxicity		After PDT		
Bacteria:	MIC (μM)	MBC (μM)	Irradiance (J·cm^−2^)	MIC (μM)	MBC (μM)
*Escherichia coli*	>200	>200	2 × 50	>200	>200
*Pseudomonas aeruginosa*	>200	>200	2 × 20	>200	>200
*Staphylococcus aureus*	>200	>200	2 × 10	0.78	0.78
*Staphylococcus epidermidis*	>200	>200	2 × 10	<0.2	<0.2
*Enterococcus faecalis*	>200	>200	2 × 20	0.39	3.13
*Enterococcus faecium*	>200	>200	2 × 20	3.13	3.13
*MRSA*	>200	>200	2 × 10	12.5	12.5
*Bacillus subtilis*	>200	>200	2 × 10	3.13	3.13

**Table 4:** MIC and MBC values of PdTPPS_4_ on different pathogens without irradiation (dark toxicity) and after PDT. The color scale indicates the relative therapeutic effectiveness within the tested concentration range, with green corresponding to the highest effectiveness and red corresponding to the lowest effectiveness.

Bacteria	Dark Toxicity		After PDT		
	MIC (μM)	MBC (μM)	Irradiance (J·cm^−2^)	MIC (μM)	MBC (μM)
*Escherichia coli*	>200	>200	2 × 50	>200	>200
*Pseudomonas aeruginosa*	>200	>200	2 × 20	50	>200
*Staphylococcus aureus*	200	200	2 × 10	12.5	12.5
*Staphylococcus epidermidis*	50	200	2 × 10	<0.2	<0.2
*Enterococcus faecalis*	>200	>200	2 × 20	25	>200
*Enterococcus faecium*	200	>200	2 × 20	25	25
*MRSA*	>200	>200	2 × 10	12.5	50
*Bacillus subtilis*	>200	>200	2 × 10	>200	>200

**Table 5:** MIC and MBC values of MB on different pathogens without irradiation (dark toxicity) and after PDT. The color scale indicates the relative therapeutic effectiveness within the tested concentration range, with green corresponding to the highest effectiveness and red corresponding to the lowest effectiveness.

Bacteria	Dark Toxicity		After PDT		
	MIC (μM)	MBC (μM)	Irradiance (J·cm^−2^)	MIC (μM)	MBC (μM)
*Escherichia coli*	>200	>200	2 × 50	6.25	6.25
*Pseudomonas aeruginosa*	>200	>200	2 × 20	1.56	3.13
*Staphylococcus aureus*	100	>200	2 × 10	0.2	0.2
*Staphylococcus epidermidis*	12.50	50	2 × 10	0.39	0.39
*Enterococcus faecalis*	200	200	2 × 20	6.25	6.25
*Enterococcus faecium*	200	>200	2 × 20	12.50	25
*MRSA*	100	200	2 × 10	0.39	0.39
*Bacillus subtilis*	50	100	2 × 10	3.13	3.13

**Table 6:** MIC and MBC values of ZnPCS_2_ on different pathogens without irradiation (dark toxicity) and after PDT. The color scale indicates the relative therapeutic effectiveness within the tested concentration range, with green corresponding to the highest effectiveness and red corresponding to the lowest effectiveness.

Bacteria	Dark Toxicity		After PDT		
	MIC (μM)	MBC (μM)	Irradiance (J·cm^−2^)	MIC (μM)	MBC (μM)
*Escherichia coli*	>200	>200	2 × 50	100	100
*Pseudomonas aeruginosa*	>200	>200	2 × 20	3.13	6.25
*Staphylococcus aureus*	>200	>200	2 × 10	0.78	0.78
*Staphylococcus epidermidis*	50	>200	2 × 10	0.39	0.78
*Enterococcus faecalis*	>200	>200	2 × 20	0.78	0.78
*Enterococcus faecium*	>200	>200	2 × 20	1.56	1.56
*MRSA*	100	>200	2 × 10	0.39	0.78
*Bacillus subtilis*	>200	>200	2 × 10	0.78	0.78

**Table 7:** Comparative summary of the antimicrobial efficacy of evaluated photosensitizers on Gram-positive (Gram+) and Gram-negative (Gram−) bacteria.

Photosensitizer	Chemical Class	Absorption Region	Gram−	Gram+	Overall Efficacy	Key Advantages	Limitations
TMPyP	Cationic porphyrin	Blue (~414 nm)	High	Moderate	High	Good solubility, strong effect on Gram-positive	Lower efficacy in Gram-negative
Protoporphyrin IX	Porphyrin	Blue (~409 nm)	High	Low	Moderate	High efficacy in Gram-positive, no dark toxicity	Ineffective in Gram-negative
PdTPPS_4_	Metalloporphyrin	Blue (~410 nm)	Moderate	Low	Low–Moderate	Water-soluble	Weak overall antimicrobial activity
MB	Phenothiazine	Red (~660 nm)	Very high	High	Very high	Broad-spectrum, low MIC, clinically established	Mild dark toxicity at higher concentrations
ZnPCS_2_	Phthalocyanine	Red (~630–670 nm)	Very high	High	Very high	Strong efficacy, low MIC, effective in Gram-negative	Limited prior clinical data

**Table 8:** Determination of quantitative sensitivity of resistant strains (R) to antibiotics (MIC in mg/L).

Bacterial Strain								
*Methicilin–Oxacilin Resistant Staphylococcus Aureus* 4591/A						*Enterococcus faecium 419 VanA*		
oxacilin	8	R	chloramfenikol	4		ampicilin	32	R
tetracyklin	1		kontrimoxazol	1		linezolid	0.5	
erytromycin	>4	R	klindamycin	>8	R	ciprofloxacin	>8	R
ciprofloxacin	>4	R	gentamicin	0.5		tigecyklin	0.1	
vankomycin	1		teikoplanin	1		vankomycin	>16	R
linezolid	2		tigecyklin	0.25				

## Data Availability

The original contributions presented in this study are included in the article. Further inquiries can be directed to the corresponding authors.
